# "Smart Eye Camera": An innovative technique to evaluate tear film breakup time in a murine dry eye disease model

**DOI:** 10.1371/journal.pone.0215130

**Published:** 2019-05-09

**Authors:** Eisuke Shimizu, Yoko Ogawa, Hiroyuki Yazu, Naohiko Aketa, Fan Yang, Mio Yamane, Yasunori Sato, Yutaka Kawakami, Kazuo Tsubota

**Affiliations:** 1 Department of Ophthalmology, Keio University School of Medicine, Tokyo, Japan; 2 OUI Inc., Tokyo, Japan; 3 Department of Ophthalmology, Tsurumi University School of Dental Medicine, Kanagawa, Japan; 4 Aier Eye School of Ophthalmology, Central South University, Tokyo, Japan; 5 Department of Preventive Medicine and Public Health, Biostatistics at Clinical and Translational Research Center, Keio University School of Medicine, Tokyo, Japan; 6 Division of Cellular Signaling, Institute for Advanced Medical Research, Keio University, Tokyo, Japan; University of California Berkeley, UNITED STATES

## Abstract

Tear film breakup time (TFBUT) is an essential parameter used to diagnose dry eye disease (DED). However, a robust method for examining TFBUT in murine models has yet to be established. We invented an innovative device, namely, the "Smart Eye Camera", which addresses several problems associated with existing methods and is capable of evaluating TFBUT in a murine DED model. We compared images taken by existing devices and the Smart Eye Camera in a graft-versus-host disease-related DED murine model. We observed that the quality of the images obtained by the Smart Eye Camera were sufficient for practical use. Moreover, this new technique could be used to obtain measurements for several consecutive ocular phenotypes in a variety of environments. Here, we demonstrate the effectiveness of our new invention in the examination of ocular phenotypes, including TFBUT in a murine model. We highlight the potential for future translational studies adopting the Smart Eye Camera in clinical settings.

## Introduction

Dry eye disease (DED) is a common ocular disease and a major reason for visits to ophthalmologists. It is reported that 7.4–33.4% of the worldwide population has been diagnosed with DED [1], comprising an estimated 560 million to 2.54 billion DED patients [2]. Recently, an article by the International Dry Eye Work Shop on the role of the tear film in DED was published [3]. DED is characterized by the loss of tear volume, rapid breakup of the tear film, and evaporation of tears. It is proposed that tear film breakup time (TFBUT) is the one of the core objective findings in DED diagnosis, and it induces declines in visual performance and optical quality [3]. For example, a previous article reports that DED decreases human annual labor productivity by $6,160 per year per capita [4]. Because of the increased incidence of this disease in Asia, the diagnostic criteria of DED were renewed by the Asia Dry Eye Society [5]. The renewed criteria highlight an essential role of TFBUT assessment and defined TFBUT as the most important objective phenotype in DED patients. Despite the importance of TFBUT assessment in humans, a robust method of measuring TFBUT is not established in murine DED models.

There are several reasons that render TFBUT evaluation uncommon in mice. First, the mouse cornea is only 2–3 mm in width, and its small size makes it challenging to evaluate due to the difficulty of adjusting the instrument focus [6]. Second, there are issues with the observation devices used. The slit-lamp microscope for clinical use has been employed in the past [7]. However, it is immovable, which makes continuous observation impossible as it cannot repeatedly be moved into specific pathogen-free (SPF) environments. Other studies have used a portable hand-held slit lamp device [8, 9]. However, this device cannot record any photos, and at least three people are required to obtain the TFBUT [9], whereby the first person holds the mouse, the second person injects the fluorescein solution, and the third person uses the portable slit-lamp. Third, existing devices are expensive and have low cost-effectiveness. To circumvent these issues, there has been increased usage of tear secretion (TS) and corneal fluorescein score (CFS) for diagnosis in murine models of DED [10, 11]. Nevertheless, an optimal method for measuring the most important ocular phenotype in murine DED models has yet to be established. Moreover, in basic *in vivo* studies, continuous observation is necessary to evaluate the alteration of phenotypes. Thus, we believe that a new and inexpensive device that allows easy evaluation of TFBUT in murine DED models will have great value.

**Fig 1:**
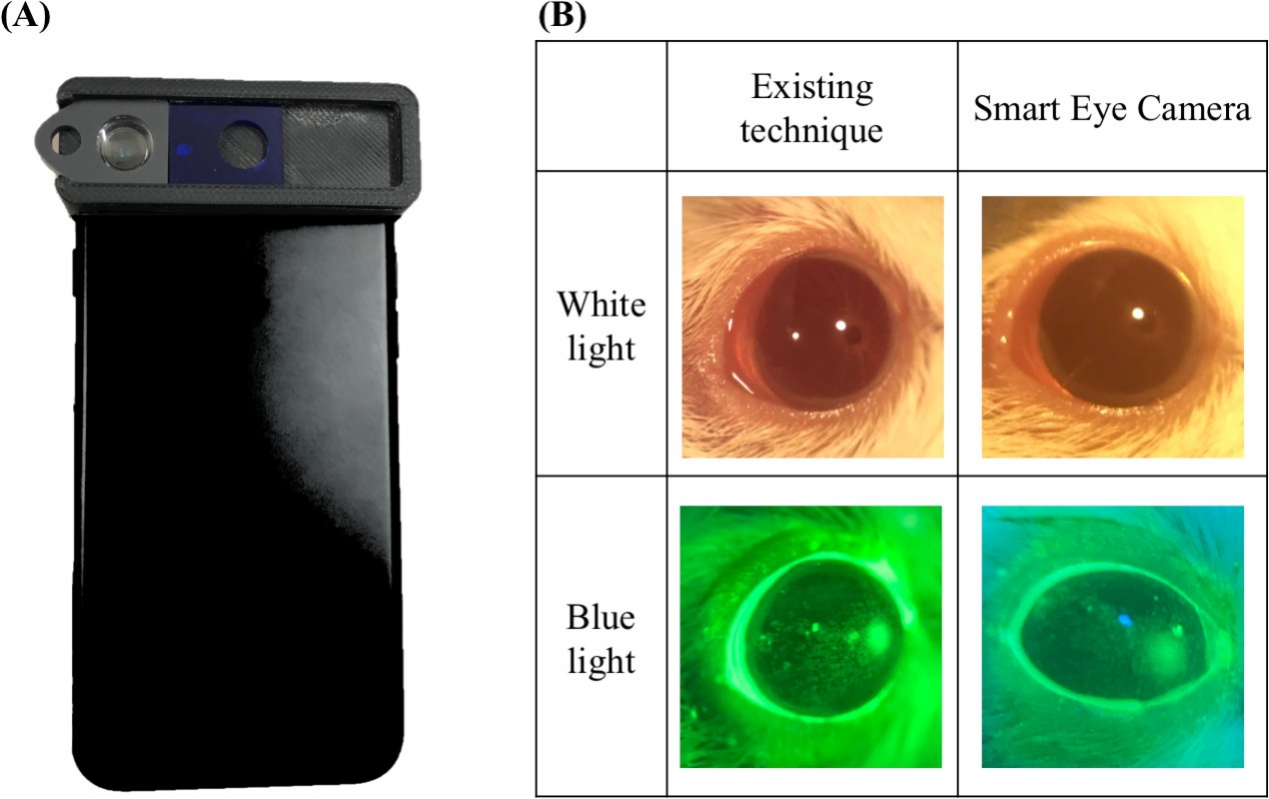
The appearance of Smart Eye Camera and representative photo. (A) This invention is a portable attachment for smartphones. The current model was designed for the iPhone 7, which contains a convex macro lens and blue filter. (B) The upper row shows representative photos of the eye to which white light has been applied. The lower row shows fluorescein stained images. The left column images were taken by the existing techniques. The right column images were taken by the Smart Eye Camera.

We therefore invented a portable attachment, referred to as the “Smart Eye Camera” (Fig 1). This device can connect to any smartphone and take images and videos of mouse eyes, and it has the ability to resolve many of the issues mentioned above. For instance, it is portable and easily operated by a single person and has an adjustable focus and low manufacturing cost. Therefore, we hypothesized that the Smart Eye Camera could obtain several ocular phenotype measurements, such as TFBUT and CFS in a murine DED model (Fig 1). If the functionality of this new invention is proven successful for mouse DED models, there is a possibility that it could be applied in clinical practice. To assess the function of the Smart Eye Camera, we used an established graft-versus-host disease (GVHD)-related murine model of DED in conjunction with our device to evaluate ocular surface conditions, including TFBUT and CFS.

The purpose of this study was to examine the utility of the Smart Eye Camera in a murine GVHD-related DED model and to establish its effectiveness in order to provide a basis for further research involving its use in clinical practice.

## Materials and methods

### The Smart Eye Camera

The Smart Eye Camera is an attachable device that connects to smartphones (Fig 1). The frame was produced by a 3D printer (MakerBot Replicator 5th Generation, MakerBot Industries, LLC, Japan) using polylactic resin. It has a removable convex macro lens (focal length: 10–30 mm, magnification: ×20) above the lens of the smartphone to adjust the focus. In addition, it contains a removable acrylic resin blue filter (PGZ 302K 302, Kuraray Co, LTD. Japan) above the light source of the smartphone to convert the white light to 488 nm wavelength blue light. Furthermore, there is a removable 525–550 nm wavelength band-pass filter above the convex macro lens (CC-G50, Fujifilm Corporation, Japan), which can emphasize the excitation light stimulated by the blue light and the fluorescein solution. For the current study, an iPhone 7 (Apple Inc., Cupertino, CA, USA) was used as the camera and light source.

The illumination of the existing light source is 14,000–30,000 lux with no blue filter and 1,500–2,600 lux with the blue filter. The illumination of the smartphone used in the current study is 8,000 lux with no blue filter and 2,000 lux with the blue filter (observed by using an LX-1010B digital lux illuminator, Zhangzhou WeiHua Electronic Co. Ltd., China).

### Existing instruments

We used an existing microscope (microscope: SZ61, camera: DP70, light source: LG-PS2; Olympus Corporation, Tokyo, Japan) and portable slit-lamp microscope (SL-15, Kowa Company Limited, Tokyo, Japan) for comparing the devices. These instruments are widely used for evaluating ocular phenotypes in murine DED models [12].

### Murine GVHD-related DED model

All experimental procedures were approved by the Keio University Institutional Animal Care and Use Committee. Our protocols for animal experiments were in accordance with the Institutional Guidelines on Animal Experimentation at Keio University (#09152). We also followed the ARVO Statement for the Use of Animals in Ophthalmic and Vision Research [13]. For the DED mouse model, which is a well-established model, we chose Zhang's method [14], which reproduces the phenotype of GVHD-related DED as observed in clinical cases [12, 15]. This GVHD-related DED animal model typically has an approximately 90% success rate [12, 15–18]. B10.D2 and BALB/cCrSlc (BALB/c) mice (7 weeks of age) were purchased from Sankyo Laboratory Inc. (Tokyo, Japan). All mice were kept at the Laboratory Animal Center, Keio University School of Medicine (Tokyo, Japan) in an SPF environment in which the temperature and humidity were controlled in the range of 23–26°C and 60–70%, respectively. After adaptation to the SPF environment for a week, the mice were divided into three groups (five mice per group). For the DED (GVHD model) group, allogeneic bone marrow transplantation (BMT) was conducted using 8 weeks of age male B10.D2 and female BALB/c mice for donors and recipients, as previously reported [12, 15–18]. For the negative control (non-GVHD), syngeneic BMT was conducted by transplanting donor cells from male BALB/c mice to female BALB/c mice. These recipient mice were irradiated with 700 cGy using a Gammacel 137Cs source (Hitachi Medico Ltd., Tokyo, Japan) 6 h before BMT, then the donor cells were injected via a tail vein injection. For healthy controls (normal control), age-matched female BALB/c mice were selected; no intervention was administered to this group.

### Study design

Three mouse models were used for the comparison, as described above (GVHD, non-GVHD, and normal control). The required sample size was difficult to calculate because there were no previous reference values. Therefore, similar evidence [8] was selected for the calculation of the sample size: it was determined that 15 mice were needed in total for this pilot investigation. The DED phenotype in this murine GVHD-related DED model appears 3 weeks after BMT [12]. Therefore, in addition to our standard protocol, we collected several ocular phenotypes, including body weight, TFBUT, CFS, and TS, once a week, from before BMT (8 weeks of age) until 12 weeks of age (5 time points). The phenotypes were evaluated according to the methods described below. All of the phenotypes were evaluated without any anesthesia. Notably, consecutive TFBUT and CFS were only evaluated by the Smart Eye Camera because it was possible to sterilize and bring the instrument into the SPF environment. To compare the validity between existing techniques and the Smart Eye Camera, TFBUT and CFS were both evaluated when the mice were 12 weeks of age, after the mice were removed from the SPF environment because the other equipment could not be brought into the SPF room. To control the order effect of the two instruments, we changed their order randomly by using a table of random digits. All data captured by the Smart Eye Camera were manually transferred to an iMac (Apple Inc.) via Bluetooth and converted to mp4 video data for safe storage. Before the evaluation by three ophthalmology specialists (ES, YO, and HY), data were individually masked.

### Evaluation of tear film breakup time

Tear film stability was measured using the TFBUT. The observer held the mice in one hand, and then instilled 1 μl of 0.5% sodium fluorescein into the conjunctival sac [9, 19] using a micropipette (Continuously Adjustable Air Displacement Pipette, P2. F144801; Gilson Inc., Villiers le Bel, France). After the three blinks, the observer used the Smart Eye Camera in his right hand and recorded a movie of the eyes with the iPhone’s video recording application. The resolution of the movie was 4K with a 30-fps frame rate. To compare the new and existing techniques, the TFBUT was obtained by an existing device and was evaluated according to a method described previously [9].

### Evaluation of corneal fluorescein score

Corneal epithelial damage was assessed using the CFS, which was assessed 90 s after fluorescein instillation. Each cornea was divided into four quadrants and scored individually. The CFS was calculated using a 4-point scale: 0, absent; 1, slightly punctate staining with < 30 spots; 2, punctate staining with > 30 spots but not diffuse; 3, severe diffuse staining but no positive plaques; and 4, positive fluorescein plaques. The four quadrants’ scores were summed to produce a final grade (0 to 16 points) [7, 19].

### Evaluation of tear secretion

TS was measured using a modified Schirmer’s test [20]. A phenol red thread (Zone-Quick; AYUMI Pharmaceutical Corporation, Tokyo, Japan) was placed on the temporal side of the upper eyelid margin for 15 s. The length of the moistened area from the edge was measured to within 0.5 mm [12, 21].

### Statistical and data analysis

**Fig 2:**
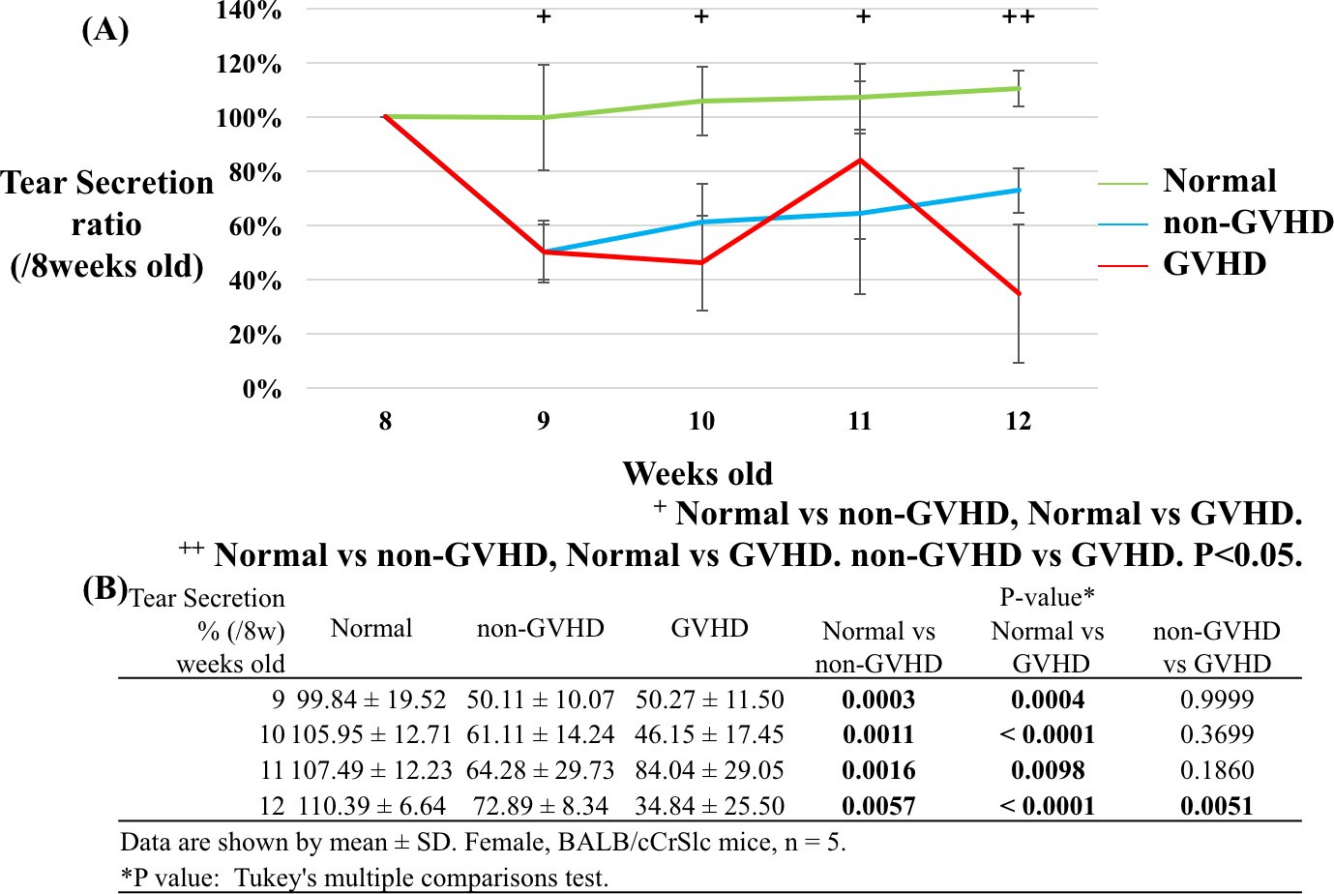
Changes in the tear secretion ratio compared to the baseline. (A) Consecutive changes in tear secretion (TS) according to group (green: normal group, blue: non-GVHD group, and red: GVHD group). (B) The table shows the TS ratio compared to the baseline (8 weeks of age). Significant differences in TS were observed between the normal and the non-GVHD group and between the normal and the GVHD group from 9 to 12 weeks of age. Furthermore, at 12 weeks of age, TS was significantly decreased in the GVHD group compared to that in the non-GVHD group. N = 5 per group. P < 0.05, Tukey's multiple comparison test.

**Fig 3:**
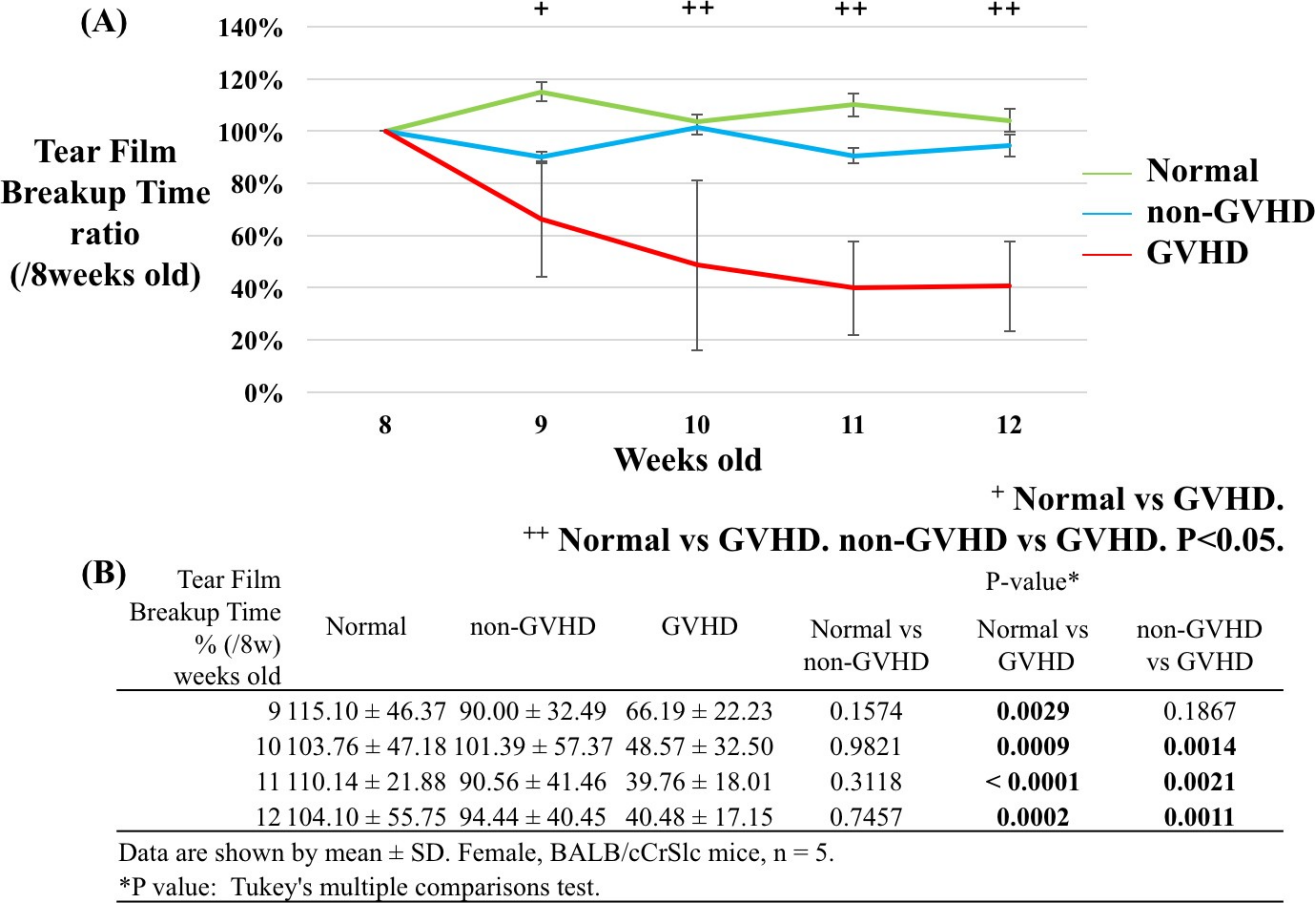
Changes in the tear film breakup time ratio compared to the baseline recorded by the Smart Eye Camera. (A) consecutive tear film breakup time (TFBUT) according to group (green: normal group, blue: non-GVHD group, and red: GVHD group). (B) The table shows the TFBUT ratio compared to that at the baseline (8 weeks of age). Significant differences in TFBUT were observed between the normal and GVHD groups from 9 to 12 weeks of age and between the non-GVHD and GVHD groups from 10 to 12 weeks of age. TFBUT was evaluated in the right eye (n = 5 per group. P < 0.05, Tukey's multiple comparison test).

**Fig 4:**
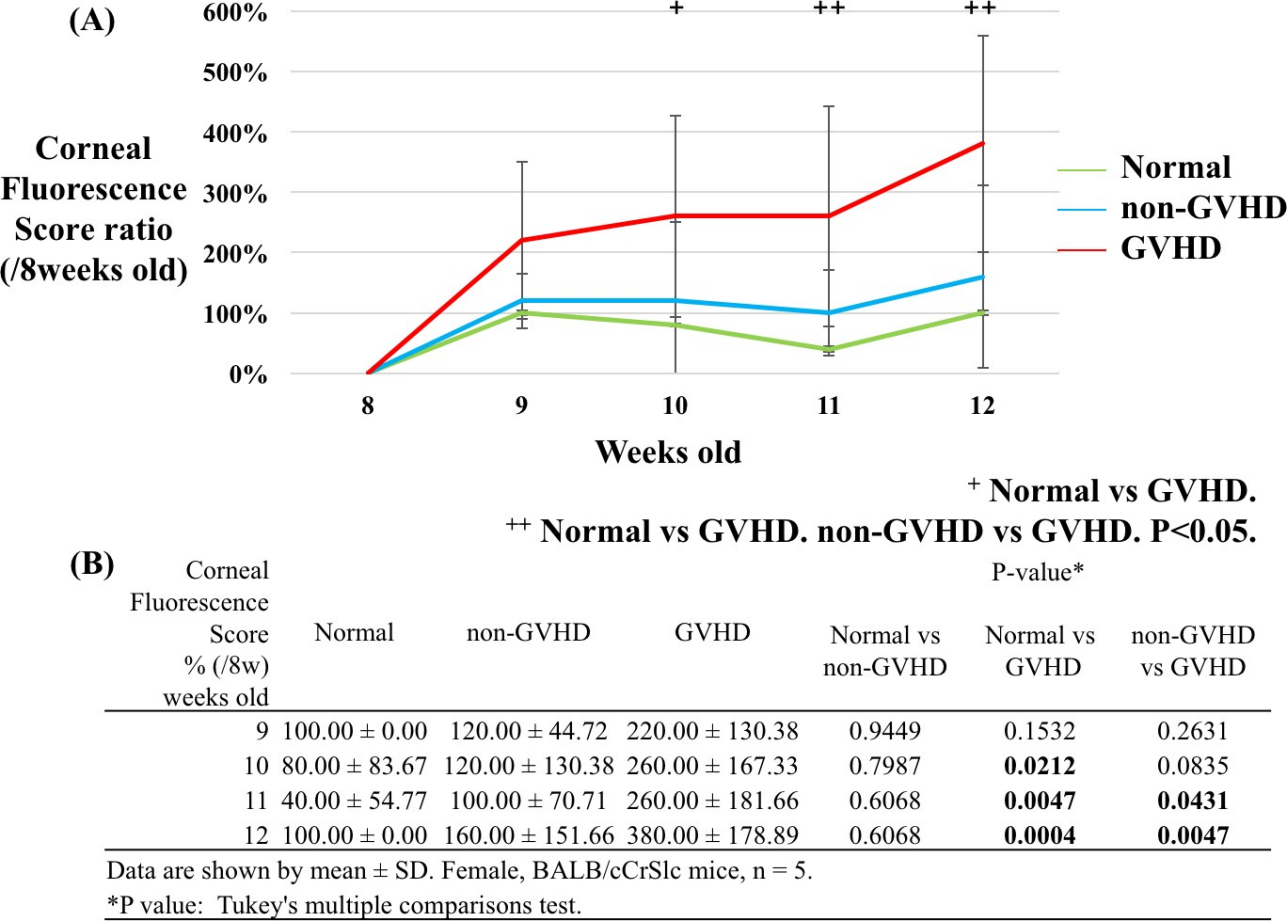
Changes in the corneal fluorescein score ratio compared to the baseline recorded by the Smart Eye Camera. (A) consecutive values for the corneal fluorescein score (CFS) according to group (green: normal group, blue: non-GVHD group, and red: GVHD group). (B) The table shows the CFS ratio compared to that at the baseline (8 weeks of age). Significant differences in CFS were observed between the normal and GVHD groups from 10 to 12 weeks of age and between the non-GVHD and GVHD groups at 11 and 12 weeks of age. CFS was evaluated in the right eye (n = 5 per group. P < 0.05, Tukey's multiple comparison test).

**Fig 5:**
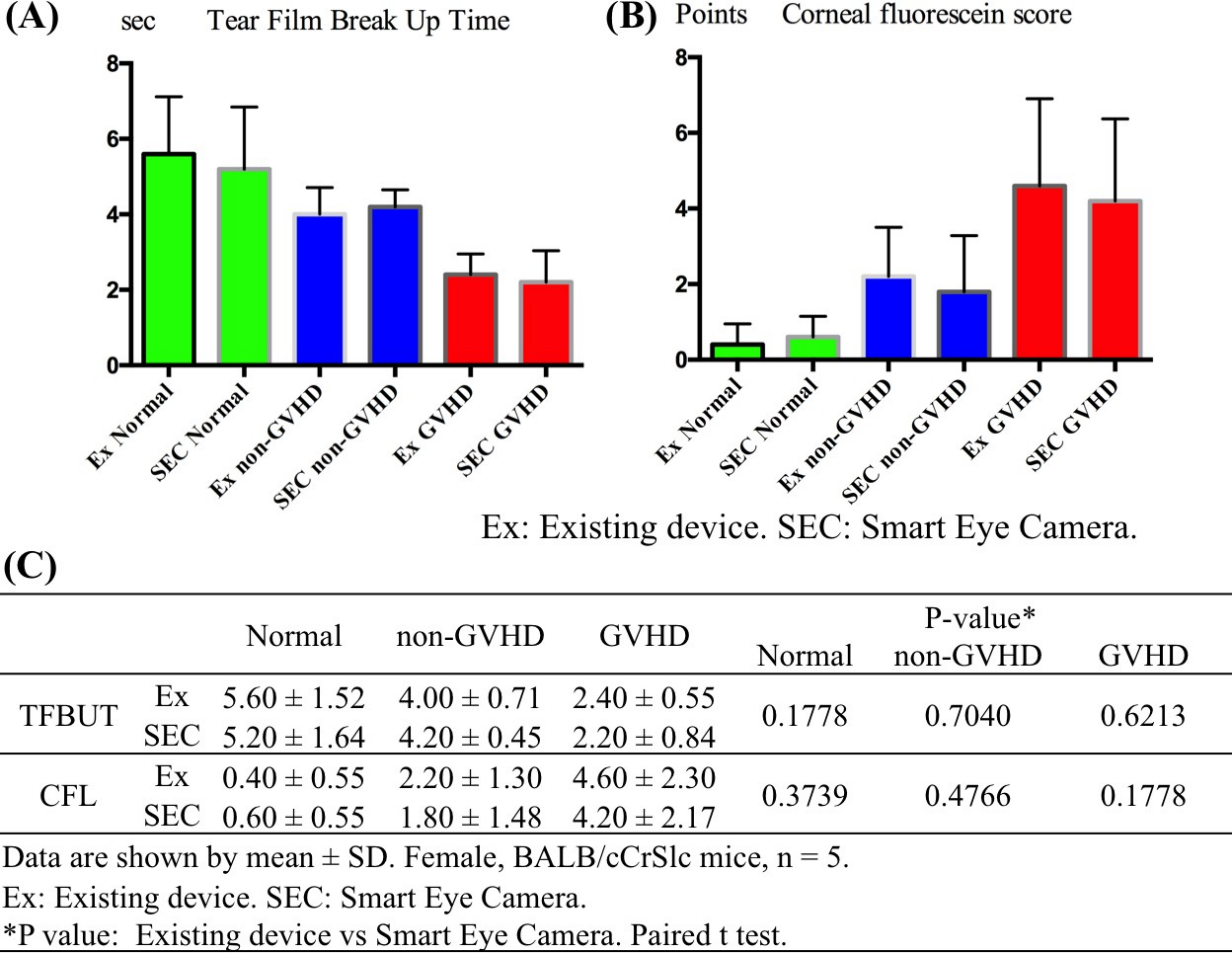
Comparison between the new and existing techniques. (A) The left graph shows TFBUT and (B) the right graph shows CFS results (green: normal group, blue: non-GVHD group, and red: GVHD group). In each graph, the two bars side by side demonstrate that there were no significant differences between the use of existing technologies and the Smart Eye Camera in the normal, non-GVHD, and GVHD groups (TFBUT and CFS. all P > 0.05). (C) The table shows the numerical values (n = 5; paired t-test).

Data were analyzed using Prism software (ver. 6.04 for Mac; GraphPad Software, Inc., San Diego, CA, USA) and SPSS (ver. 24 for Mac; IBM Corp., Armonk, NY, USA). To assess normality, quantile-quantile (q-q) plots were made using the values of TFBUT, CFS, TS, and body weight at baseline (8 weeks of age). These points followed a strongly linear pattern, suggesting that the data were normally distributed (S1 Fig). The consecutive phenotypes, including TFBUT, CFS, and TS, and body weight were analyzed as the amount of change from baseline which makes the data distributed as a normal standard. Next, a repeated measures ANOVA was performed. To compare differences in the amount of change from the baseline and the numeric values in TFBUT, CFS, TS, and body weight between the three groups, Tukey's multiple comparisons test was performed (Figs 2, 3, 4 and S2 and S5 Figs). To compare differences between the results from the existing technique and those from the Smart Eye Camera, a paired t-test was performed (Fig 5). To compare differences in TFBUT obtained by Smart Eye Camera and evaluated by three different ophthalmology specialists (ES, YO, and HY), Tukey's multiple comparisons test was performed (S3 Fig).

**Fig 6:**
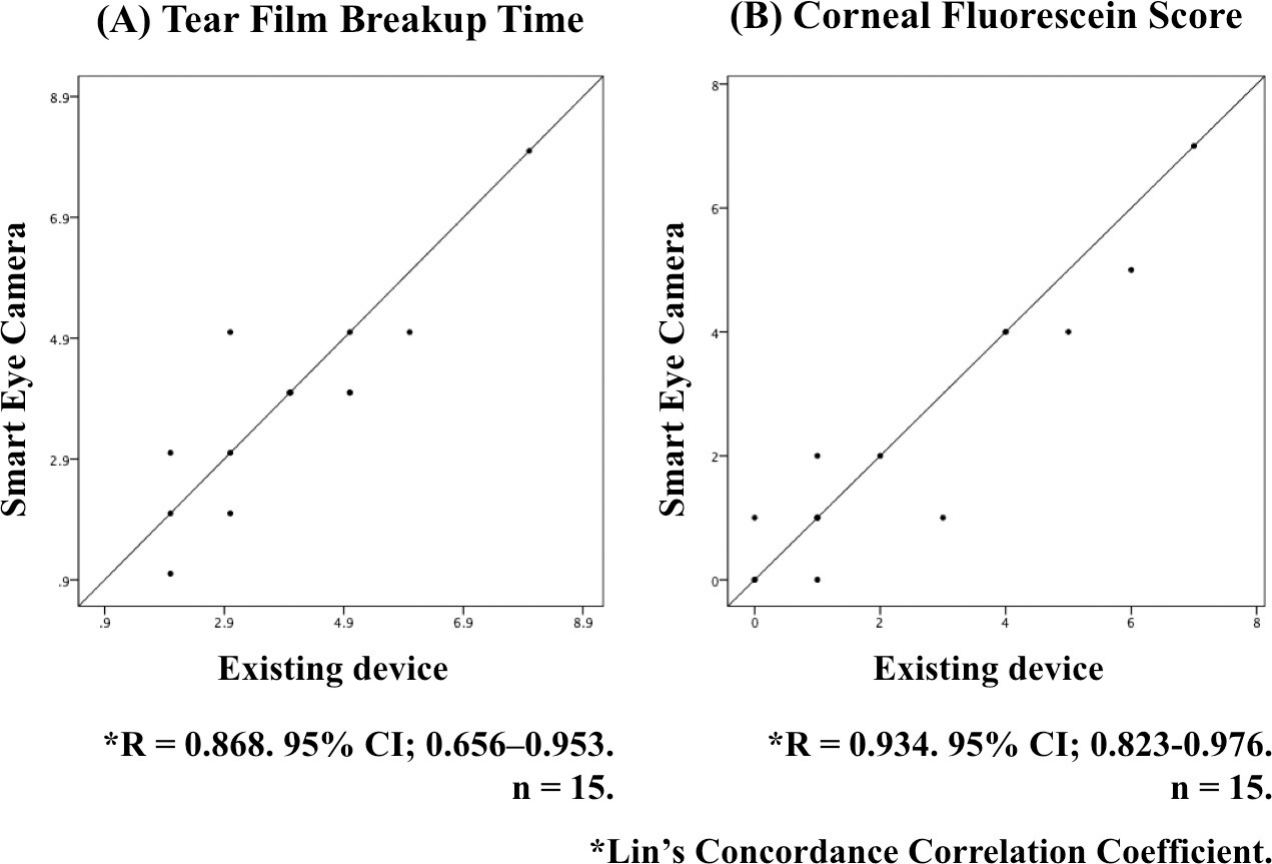
Correlation between the results of the Smart Eye Camera and existing device. (A) The left graph shows TFBUT and (B) the right graph shows CFS results. In each graph, the Y axis shows the numbers evaluated by the Smart Eye Camera and X axis shows evaluations by the existing device. Strong correlations were observed in TFBUT (R = 0.868, 95% CI; 0.656–0.953) and CFS (R = 0.934, 95% CI; 0.823–0.976); n = 15; Lin’s Concordance Correlation Coefficient.

To assess the correlation between the Smart Eye Camera and the existing technique for the TFBUT and CFS evaluations, Lin’s Concordance Correlation Coefficient was selected (Fig 6, and S4 Fig). The data are expressed as the mean ± standard deviation (SD). A P-value of < 0.05 was considered statistically significant.

## Results

### Production of GVHD-related DED model

For the first time, we confirmed the success with producing the GVHD-related DED model by evaluation of body weights and TS [12]. There was no difference between the normal, non-GVHD, and GVHD groups at the baseline as body weights were adjusted for each group at the beginning of the experiment. However, the body weight ratio compared to the baseline was significantly decreased in the non-GVHD and GVHD groups compared to that in the normal group at 9 to 12 weeks of age (S2 Fig; 9 weeks of age, P < 0.0001 and P < 0.0001; 10 weeks of age, P < 0.0001 and P < 0.0001; 11 weeks of age, P = 0.0222 and P < 0.0001; 12 weeks of age, P = 0.0142 and P < 0.0001; normal vs non-GVHD and normal vs GVHD, respectively). In addition, body weight was significantly decreased in the GVHD group compared with that in the non-GVHD group at 11 and 12 weeks of age (P = 0.0069 and 0.0003 at 11 and 12 weeks of age, respectively). Before BMT, the TS ratio was not significantly different from that at the baseline. However, these values were significantly decreased in the non-GVHD and GVHD groups compared with the values for the normal group at 9 to 12 weeks of age (Fig 2; 9 weeks of age, P = 0.0003 and P = 0.0004; 10 weeks of age, P = 0.0011 and P < 0.0001; 11 weeks of age, P = 0.0016 and P = 0.0098; 12 weeks of age, P = 0.0057 and P < 0.0001; normal vs non-GVHD and normal vs GVHD, respectively). Furthermore, the amount of TS in the GVHD group was significantly lower compared to that in the non-GVHD group at 12 weeks of age (Fig 2; 72.89 ± 8.34 vs 34.84 ± 25.50, non-GVHD vs GVHD respectively; P = 0.0051).

### Consecutive tear film breakup time measurement by the Smart Eye Camera

**Fig 7:**
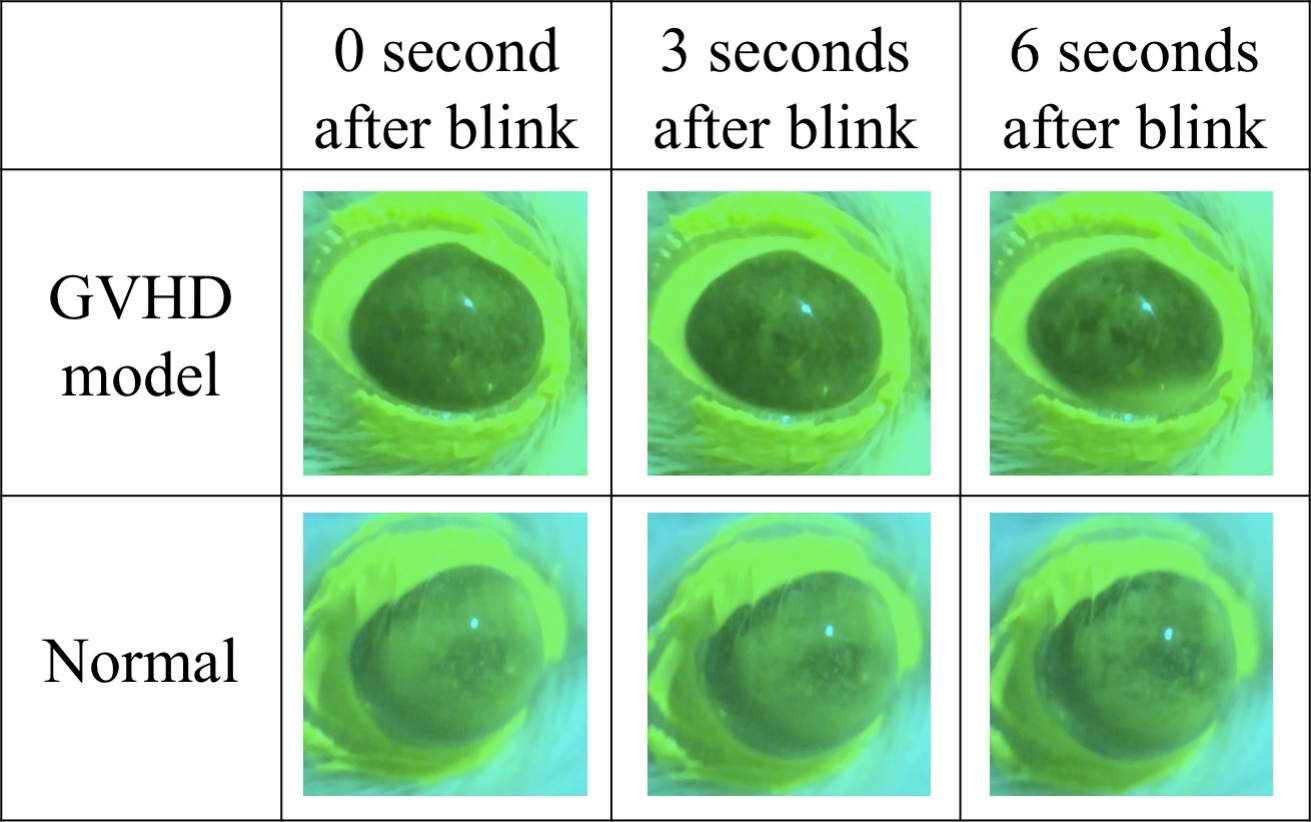
Tear film breakup pattern using the Smart Eye Camera. The figure shows a set of continuous photos taken by the Smart Eye Camera. The upper row shows images from a GVHD-related dry eye disease model mouse. The tear film was breaking up in 3 s after blinking (Tear Film Breakup Time; TFBUT = 3 s). The lower row shows the normal mouse. The tear film was stabilized in 3 s and breaking up in 6 s after blinking (TFBUT = 6 s). Photos were of the right eye in 12 weeks of age.

Fig 3 shows consecutive TFBUT ratios compared to the baseline as recorded by the Smart Eye Camera. No difference was observed between the three groups at the baseline. However, TFBUT was significantly decreased in the GVHD group compared to that in the normal group at 9 to 12 weeks of age (P = 0.0029, 0.0009, < 0.0001 and 0.0002 at 9, 10, 11, and 12 weeks of age, respectively). In addition, TFBUT decreased in the GVHD group compared to that in the non-GVHD group at 10 to 12 weeks of age (P = 0.0014, 0.0021, and 0.0011 at 10, 11, and 12 weeks of age, respectively). Fig 7 shows photos of consecutive tear films stained by fluorescein solution. A tear film breakup was observed.

### Consecutive corneal fluorescein score by the Smart Eye Camera

Fig 4 shows the consecutive corneal fluorescein score (CFS) ratios compared to those at the baseline, recorded by the Smart Eye Camera. No differences were observed between the three groups at the baseline. However, CFS was significantly increased in the GVHD group compared to that in the normal group from 10 to 12 weeks of age (P = 0.0212, 0.0047, and 0.0004 at 10, 11, and 12 weeks of age, respectively). Moreover, CFS was lower in the GVHD group compared to that in the non-GVHD group at 11 and 12 weeks of age (P = 0.0431, and 0.0047 at 11, and 12 weeks of age, respectively).

### Comparison between the new and existing technique

To compare the performance of our new invention and existing technologies, TFBUT and CFS were evaluated using both systems at the same time points. The TFBUT results from the Smart Eye Camera and the portable slit-lamp microscope showed a significant correlation (Fig 6; R = 0.868. 95% CI; 0.656–0.953; Lin’s Concordance Correlation Coefficient). Additionally, the CFS results from the Smart Eye Camera and the existing microscope showed a significant correlation (Fig 6; R = 0.934. 95% CI; 0.823–0.976; Lin’s Concordance Correlation Coefficient). Moreover, no differences were observed in the numeric values obtained for TFBUT and CFS between the Smart Eye Camera and the existing device (Fig 5; TFBUT. P = 0.1778, 0.7040, 0.6213. CFS. P = 0.3739, 0.4766, 0.1778; normal control, non-GVHD group, and GVHD group, respectively; paired t-test).

### Evaluation by multiple observers and the difference between right and left eyes

To assess the repeatability of measurements obtained with the Smart Eye Camera, we compared the results of TFBUT in 12-week-old mice evaluated by the three different ophthalmology specialists. There were no statistically significant differences between the observers at any time point (S3 Fig; 8 to 12 weeks, all P > 0.05.). Moreover, a strong correlation was observed in the ocular phenotypes between the right and the left eyes (S4 Fig; TFBUT: R = 0.714 95% CI 0.353–0.890 and CFS: R = 0.813 95% CI 0.539–0.931, n = 15, Lin’s Concordance Correlation Coefficient).

## Discussions

The purpose of this study was to demonstrate the applicability of our invention in a mouse model. Therefore, our first step is to confirm the success of producing the GVHD-related DED model. This model is characterized by body weight loss, shortening of TS, and exacerbation of corneal epithelitis, which is reflected by the CFS [7]. We obtained similar results in body weight [12, 22], TS [12, 14], and CFS [7] when compared to those in our previous studies–in both the difference ratios compared to those at the baseline (Fig 2, Fig 4 and S2 Fig) and in the numeric values (S5 Fig. Moreover, according to our results, TFBUT in the GVHD-related DED model was decreased when compared to that in the normal and the non-GVHD groups (Fig 3 and S5 Fig), which indicated that the TFBUT transition was similar with TS and opposite to CFS. A possible explanation of these consecutive results involves the use of irradiation before BMT and engraftment of donor cells to the recipients. Ten days after BMT, donor cells were engrafted in the recipients. After 21 days (from BMT), allogenic recipients presented with GVHD. Therefore, exposure to these conditions may shorten TFBUT and increase the CFS. Summarizing the first step, we verified the success of producing a GVHD-related DED model from our results.

Second, we evaluated the repeatability of the Smart Eye Camera using our major phenotypes TFBUT and CFS. In the baseline values, the data were normally distributed (S1 Fig). Moreover, the average values of TFBUT and CFS were 5.53 ± 2.03 s (95% CI; 4.51–6.56) and 0.74 ± 0.23 points (95% CI; 0.23–0.97), respectively, which demonstrates the repeatability of the Smart Eye Camera in terms of the TFBUT and CFS results.

Third, we assessed the reproducibility of the Smart Eye Camera by evaluating the three observers. The results showed similar findings across the different observers (S3 Fig), which demonstrates the reproducibility of the Smart Eye Camera.

Fourth, we performed a validity assessment by the comparison of the Smart Eye Camera and the existing device (portable slit-lamp or existing microscope). Our results revealed strong correlations for both TFBUT and CFS values obtained by the Smart Eye Camera and the existing devices (Fig 6). Additionally, there were no statistically significant differences between our invention and existing technologies, as demonstrated in Fig 5. Although lack of a significant difference does not necessarily demonstrate close agreement between methods, these outcomes do suggest that the Smart Eye Camera is able to obtain ocular phenotype images of quality equivalent to that of images obtained using existing devices in a murine model. Moreover, a strong correlation was observed in the TFBUT and CFS between the right and the left eyes (S4 Fig). We consider that the strong correlation between the two instruments, combined with the strong correlation between the results from both eyes demonstrates the validity of the Smart Eye Camera. Summarizing the above discussion, the applicability of the Smart Eye Camera was demonstrated by the repeatability, reproducibility, and validity assessments. Moreover, these results gathered in our study could be used to conduct formal estimation of sample sizes and more extensive validation of the invention.

Although it is possible to obtain a photo with only a smartphone camera, the eyes of the mice are too small for the focus to be adjusted correctly [6], resulting in a blurry image that makes it impossible to evaluate any ocular phenotypes. To solve this problem, the convex macro lens with a high magnification plays an essential role in enabling accurate focus adjustments on the mice eyes. Furthermore, the 488-nm wavelength of the blue light is essential for the ocular evaluations [23]. However, the light source of the smartphone is only a white light; therefore, converting the wavelength of the light is necessary. To solve this problem, we placed an acrylic resin blue filter in the device to convert the white light to blue light. Our results showed that the light source of the smartphone acquires the same intensity of the light illumination compared to that of existing devices. In addition, the illuminances were similar based on observations by the illuminator (please refer to the Materials and Methods section). Moreover, the removable 525–550 nm wavelength band-pass filter on the camera lens plays an important role to improve clarity under the fluorescence excitation spectrum. These features provide similar functionality to that of existing technologies [23], making it possible to obtain ocular phenotypes with the use of a smartphone.

The Smart Eye Camera offers several additional advantages. It is a portable attachment for smartphones, so external recording systems are unnecessary. Furthermore, the manufacturing cost is inexpensive due to the use of a 3D printer. Hong et al. created the anterior segment microscopic device using a 3D printer, which can be produced for under 15 US dollars [24]. Our estimated cost is lower as our device is made of polylactic resin and several lenses. Additionally, a single author can perform all of the measurement procedures alone. Therefore, cost-effectiveness is superior to existing technologies [9]. The material of the invention can be sterilized and brought into any environment, so evaluating consecutive ocular phenotype in murine models will become possible, as demonstrated in this study (Figs 1–4, 7 and S5 Fig). Thus, we have provided a seminal report of consecutive ocular phenotypes, including TFBUT and CFS in a GVHD-related DED murine model.

There are several limitations of this study. First, we did not show any pathological or biological phenotypes in this article. However, our model was a reproducible and well-established model involving full examination of ocular-related tissues, such as the conjunctiva, eyeball, eyelid, and lacrimal gland [12, 15–18]. Furthermore, this study aimed to elucidate the effectiveness of a newly invented device. Therefore, we focused on clinical ocular phenotypes for this study. The second limitation is that we only used a single type of smartphone (iPhone 7). Statista Inc. reports that there are 4.57 billion smartphone users worldwide in 2018 [25], but the market share by Apple Inc. is only 19.7%, and the quality of each model is different. Therefore, the repeatability of the results should be evaluated using different types of smartphones. The third limitation is that we only used the GVHD-related DED murine model, and the proper sample size was difficult to calculate. Our parametric modeling results are for exploratory purposes in this pilot study of a new device. Thus, future studies are needed to confirm the applicability of our device to different mouse models with proper sample sizes. In the murine DED model, we described that it is possible to measure ocular phenotypes by our new invention. However, our goal is to translate the use of the Smart Eye Camera to clinical settings. To expand the project to a clinical phase, further studies are essential to overcome the limitations of the current study, such as using multiple types of smartphones, application to other models with proper sample sizes, and evaluations by different observers.

## Conclusion

We invented a new device, the Smart Eye Camera, and demonstrated its applicability in a GVHD-related DED murine model. It is capable of aiding in the evaluation of consecutive ocular phenotypes such as TFBUT and CFS. However, due to the small sample size of this investigational pilot study, further studies are needed. Our future aim is to translate this novel technique into clinical use. We believe that the use of Smart Eye Camera can harness the benefits of widespread smartphone use and make a positive contribution to the healthcare industry.

## Supporting information

S1 Fig Quantile-quantile plot for the normality test. Figure shows quantile-quantile plots of the value of (A) TFBUT, (B) CFS, (C) TS, and (D) body weight at baseline (8 weeks of age). These points follow a strongly linear pattern, suggesting that the data are normally distributed.
(TIF)

S2 Fig Changes in the body weight ratio compared to the baseline. (A) consecutive body weight according to group (green: normal group, blue: non-GVHD group, and red: GVHD group). (B) The table shows the body weight ratio compared to the baseline (8 weeks of age). Significant differences in body weight were observed between the normal and GVHD groups and between the normal and non-GVHD groups from 9 to 12 weeks of age and between the non-GVHD and GVHD groups at 11 and 12 weeks of age (n = 5 per group. P < 0.05, Tukey's multiple comparison test).
(TIF)

S3 Fig Observer differences in evaluating tear film breakup time. The figure shows differences in the evaluation of tear film breakup time (TFBUT) between several observers. There are no significant differences between observers at any time point (8 to 12 weeks of age, all P > 0.05. Tukey's multiple comparison test).
(TIF)

S4 Fig Differences between right and left eyes. (A) The left graph shows tear film breakup time (TFBUT) and (B) the right graph shows the corneal fluorescein score (CFS). In each graph, the Y axis shows the value in the left eye and the X axis shows the value in the right eye. (C) The table shows the numerical values. A strong correlation was observed between the right and left eyes values (TFBUT: R = 0.714 95% CI 0.353–0.890 and CFS: R = 0.813 95% CI 0.539–0.931), n = 15, Lin’s Concordance Correlation Coefficient.
(TIF)

S5 Fig Numerical values of tear film breakup time, corneal fluorescein score, tear secretion, and body weight. (A) Consecutive transitions of tear film breakup time (TFBUT), (B) corneal fluorescein score (CFS), (C) tear secretion (TS), and (D) body weight according to group (green: normal group, blue: non-GVHD group, and red: GVHD group). Each table shows the numerical values. GVHD, graft-versus-host disease.
(TIF)
