# Supplementary figures and images for: "Smart Eye Camera": An innovative technique to evaluate tear film breakup time in a murine dry eye disease model
Source: PLoS One. 2019 May 9;14(5):e0215130. doi: 10.1371/journal.pone.0215130 (PMC6508640; doi:10.1371/journal.pone.0215130)

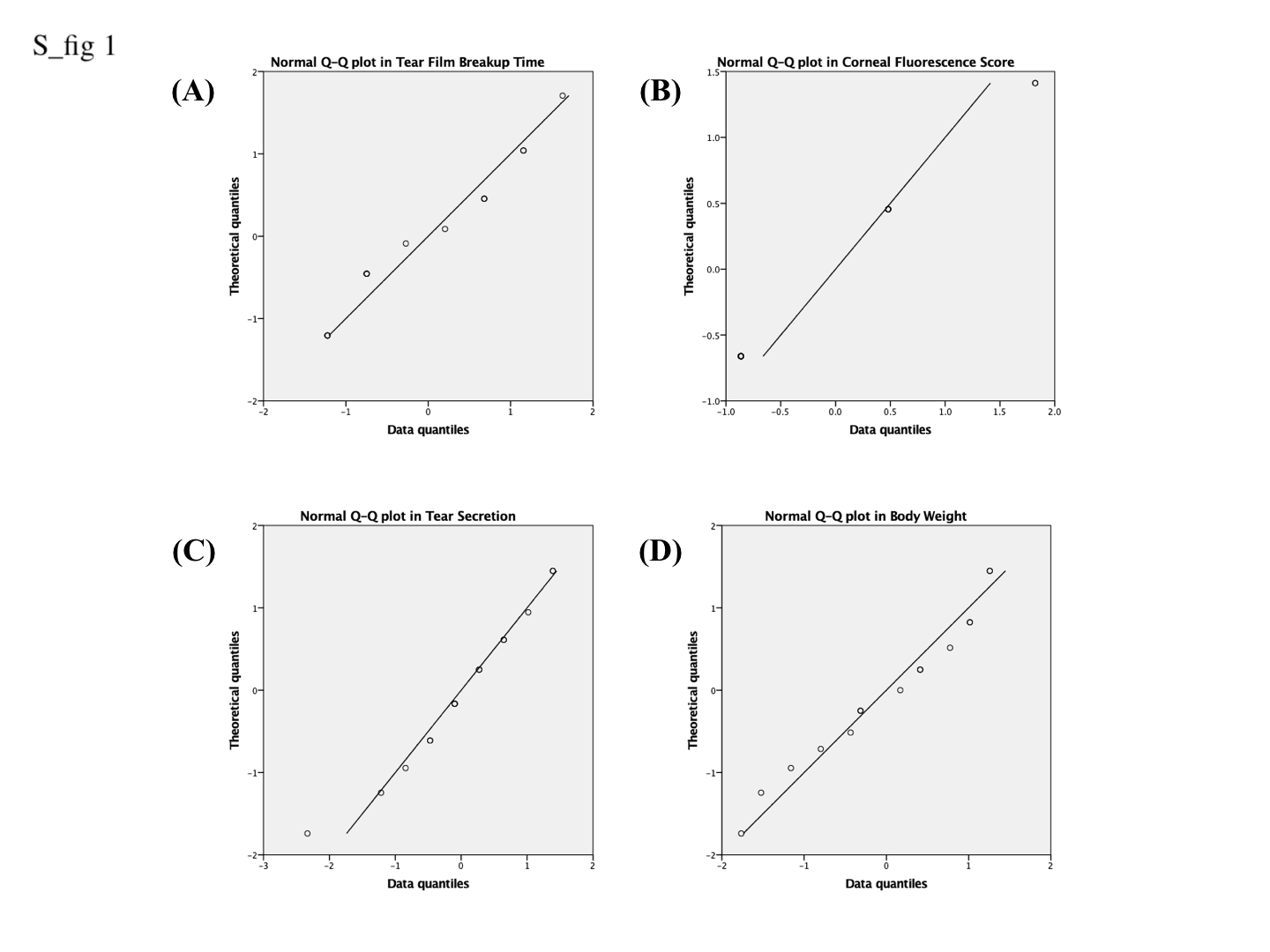

Supplement: S1 Fig — Figure shows quantile-quantile plots of the value of (A) TFBUT, (B) CFS, (C) TS, and (D) body weight at baseline (8 weeks of age). These points follow a strongly linear pattern, suggesting that the data are normally distributed. (TIF) [file pone.0215130.s001.tif]

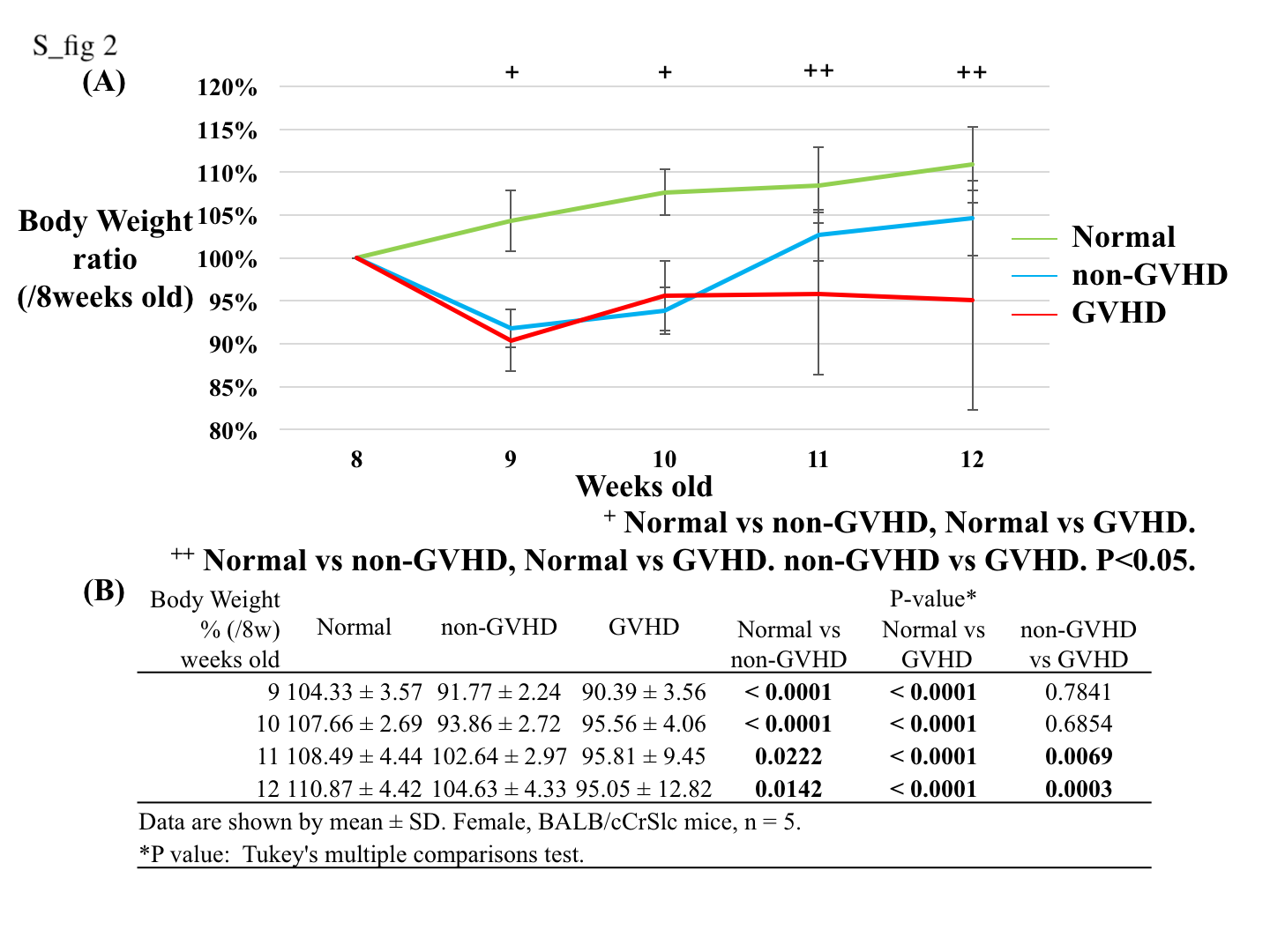

Supplement: S2 Fig — (A) consecutive body weight according to group (green: normal group, blue: non-GVHD group, and red: GVHD group). (B) The table shows the body weight ratio compared to the baseline (8 weeks of age). Significant differences in body weight were observed between the normal and GVHD groups and between the normal and non-GVHD groups from 9 to 12 weeks of age and between the non-GVHD and GVHD groups at 11 and 12 weeks of age (n = 5 per group. P < 0.05, Tukey's multiple comparison test). (TIF) [file pone.0215130.s002.tif]

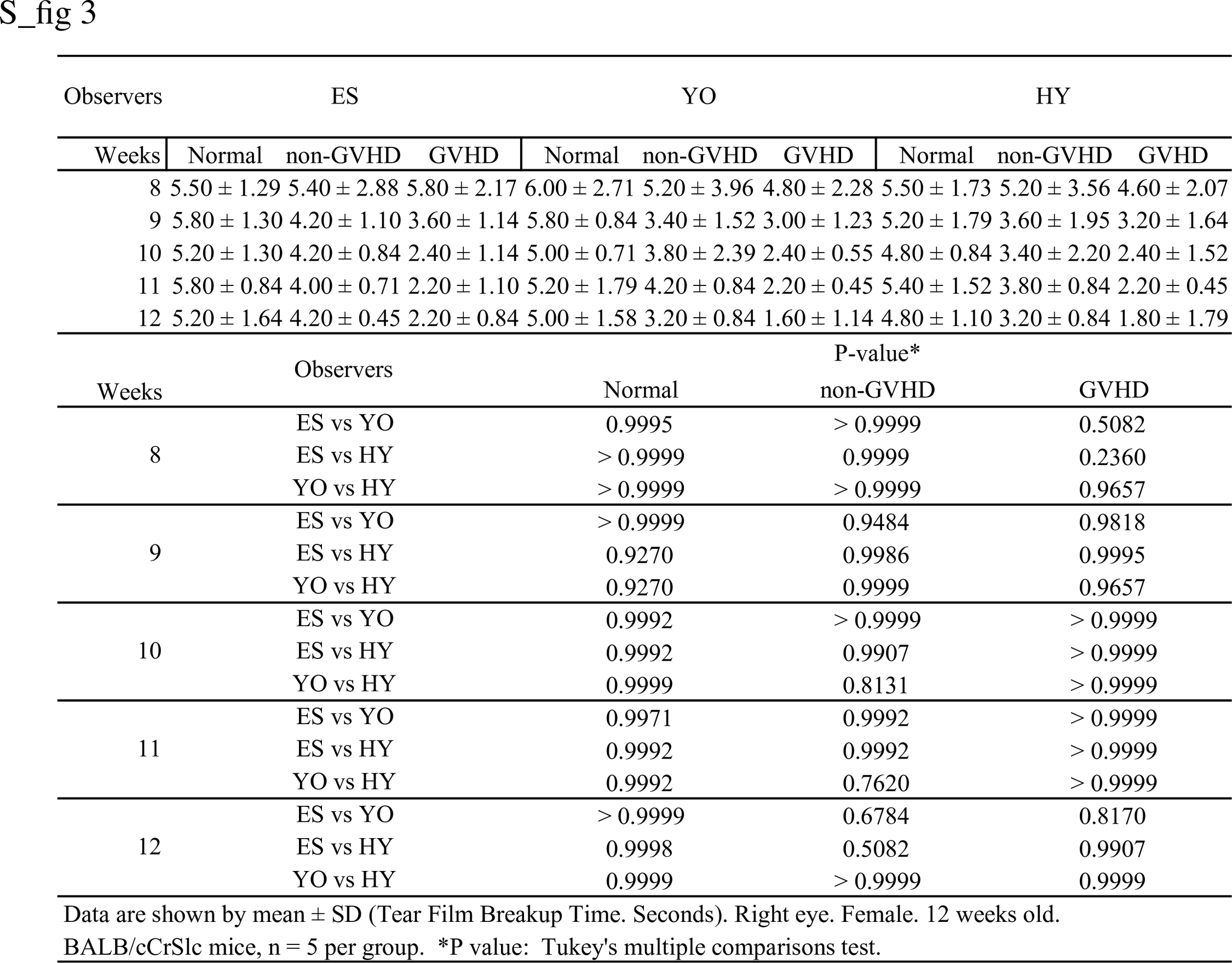

Supplement: S3 Fig — The figure shows differences in the evaluation of tear film breakup time (TFBUT) between several observers. There are no significant differences between observers at any time point (8 to 12 weeks of age, all P > 0.05. Tukey's multiple comparison test). (TIF) [file pone.0215130.s003.tif]

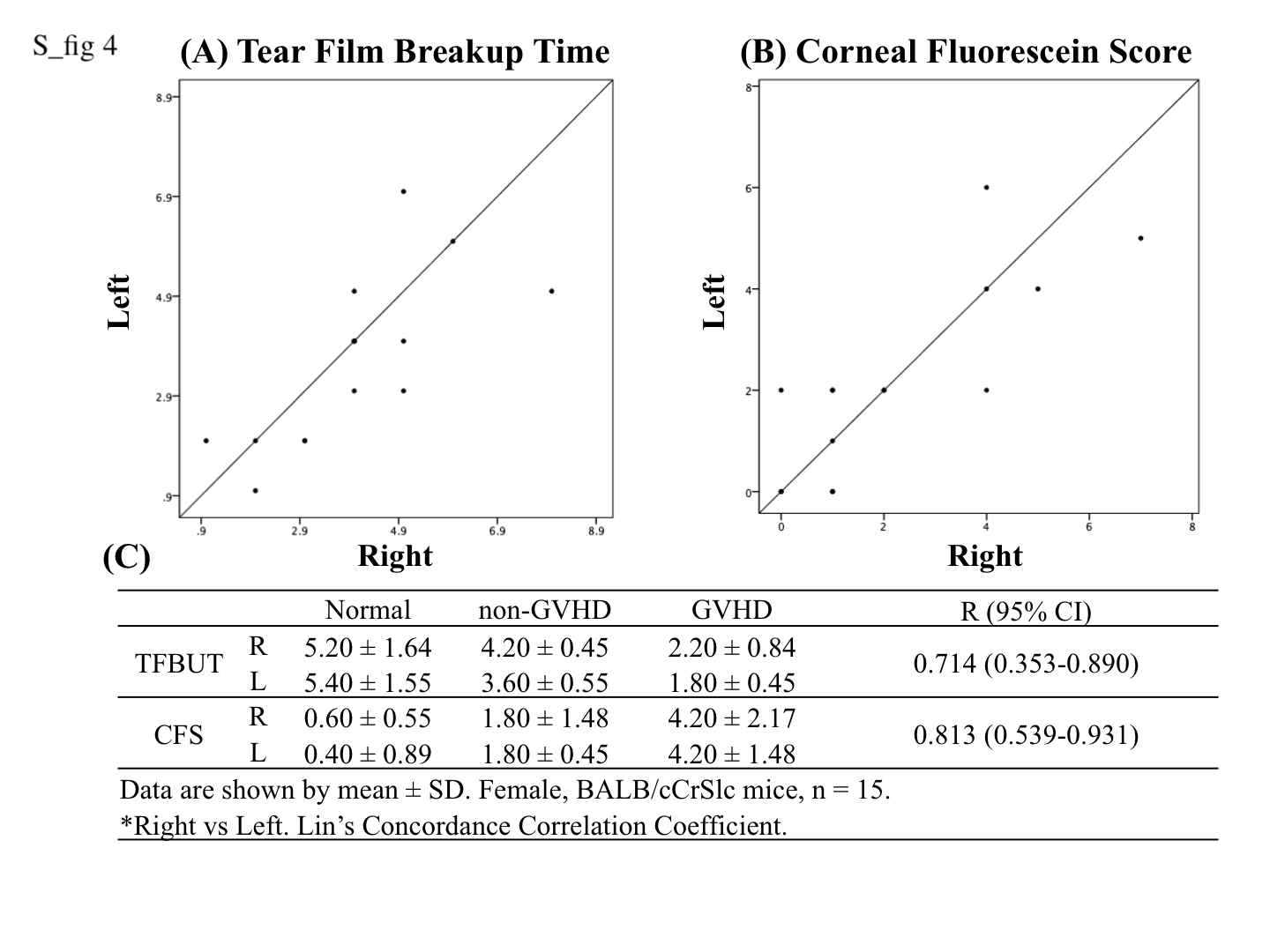

Supplement: S4 Fig — (A) The left graph shows tear film breakup time (TFBUT) and (B) the right graph shows the corneal fluorescein score (CFS). In each graph, the Y axis shows the value in the left eye and the X axis shows the value in the right eye. (C) The table shows the numerical values. A strong correlation was observed between the right and left eyes values (TFBUT: R = 0.714 95% CI 0.353–0.890 and CFS: R = 0.813 95% CI 0.539–0.931), n = 15, Lin’s Concordance Correlation Coefficient. (TIF) [file pone.0215130.s004.tif]

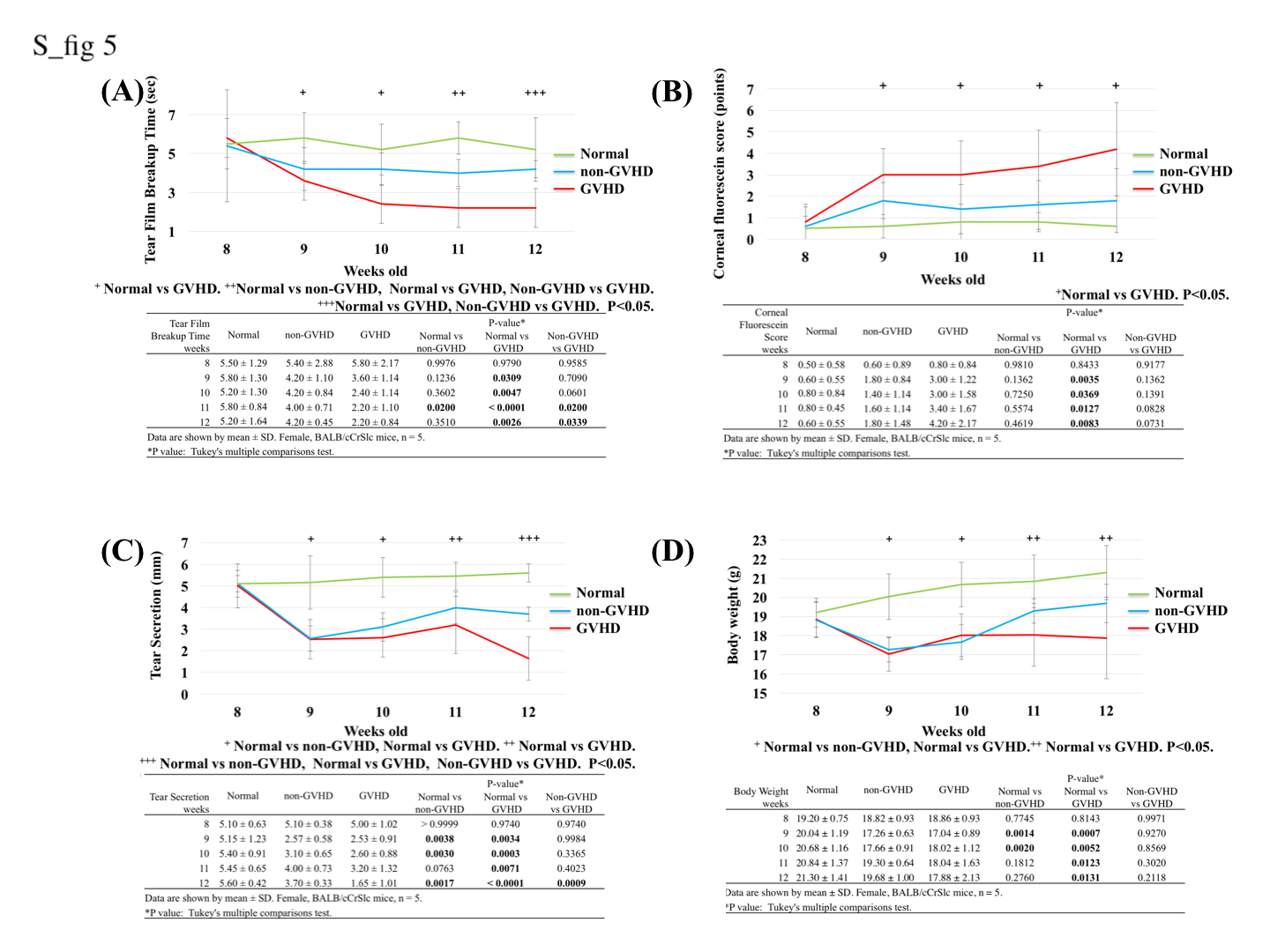

Supplement: S5 Fig — (A) Consecutive transitions of tear film breakup time (TFBUT), (B) corneal fluorescein score (CFS), (C) tear secretion (TS), and (D) body weight according to group (green: normal group, blue: non-GVHD group, and red: GVHD group). Each table shows the numerical values. GVHD, graft-versus-host disease. (TIF) [file pone.0215130.s005.tif]
